# Malaria in pregnancy control and pregnancy outcomes: a decade’s overview using Ghana’s DHIMS II data

**DOI:** 10.1186/s12936-022-04331-2

**Published:** 2022-10-27

**Authors:** Gifty Dufie Ampofo, Joseph Osarfo, Matilda Aberese-Ako, Livingstone Asem, Mildred Naa Komey, Wahjib Mohammed, Anthony Adofo Ofosu, Harry Tagbor

**Affiliations:** 1 University of Health and Allied Sciences, PMB 31, Ho, Ghana; 2 National Malaria Control Programme-Ghana Health Service, Accra, Ghana; 3 Ghana Health Service, Accra, Ghana

**Keywords:** Malaria in pregnancy, Maternal anaemia, Low birth weight, Intermittent preventive treatment of malaria, MiP interventions, Ghana

## Abstract

**Background:**

Malaria in pregnancy control interventions have been implemented through antenatal care services for more than 2 decades in Ghana. The uptake of these interventions has seen steady improvement over the years. This has occurred within the context of decreasing global trends of malaria infection confirmed by decreasing malaria in pregnancy prevalence in Ghana. However, not much is known about how these improvements in interventions uptake and reduction in malaria infection prevalence have impacted pregnancy outcomes in the country. This study aimed at describing trends of maternal anaemia and low birth weight prevalence and uptake of malaria in pregnancy control interventions over the last decade using data from Ghana’s District Health Information Management System (DHIMS II).

**Methods:**

Data from Ghana’s DHIMS II on variables of interest covering the period 2012 to 2021 was analysed descriptively using Microsoft Excel 365. Results were computed as averages and percentages and presented in tables and graphs.

**Results:**

The prevalence of maternal anaemia at booking and at term and low birth weight increased marginally from 31.0%, 25.5% and 8.5% in 2012 to 36.6%, 31.9% and 9.5% in 2021 respectively. Severe anaemia prevalence at booking and at term remained under 2% over the study period. Women making at least 4 ANC visits, receiving at least 3 doses of intermittent preventive treatment of malaria and an insecticide-treated net increased from 77.0%, 41.4% and 4.1% in 2012 to 82%, 55.0% and 93.3% in 2021, respectively. Malaria test positivity rate reduced from 54.0% to 34.3% between 2014 and 2021 while women receiving iron and folate supplementation for 3 and 6 months rose from 43.0% and 25.5% to 89.7% and 61.8%, respectively between 2017 and 2021.

**Conclusion:**

Maternal anaemia and low birth weight prevalence showed marginal upward trends over the last decade despite reduced malaria infection rate and improved uptake of malaria in pregnancy control interventions. There is room for improvement in current intervention implementation levels but the complex and multi-factorial aetiologies of maternal anaemia and low birth weight need urgent investigation and quantification to inform policy and practice.

**Supplementary Information:**

The online version contains supplementary material available at 10.1186/s12936-022-04331-2.

## Background

Malaria in Pregnancy (MiP) remains a disease of public health importance and has been on the global agenda for decades. It increases the risk of the mother and fetus to adverse pregnancy outcomes including fetal growth restrictions, low birth weight (LBW), preterm deliveries, miscarriages and stillbirths, maternal anaemia, and sometimes maternal death; women in their first and second pregnancies being particularly vulnerable [1–4]. In 2019, an estimated 11.6 million pregnancies were exposed to malaria in sub-Saharan Africa (SSA) alone which led to an estimated 822, 000 MiP-related LBW babies. Almost five in ten (49%) of these babies were born in West Africa [5].

The World Health Organization (WHO) recommends a three-pronged approach to MiP control in areas of high to moderate *Plasmodium* transmission; administration of intermittent preventive treatment using sulfadoxine-pyrimethamine (IPTp-SP), insecticide-treated net (ITN) delivery and use and prompt and effective case management [3, 6] through the antenatal care (ANC) system. A pregnant woman is currently recommended to receive at least 3 doses of IPTp-SP at monthly intervals beginning at 16 weeks as Directly Observed Treatment (DOT) [7]. In areas of hookworm infection endemicity, the WHO further recommends provision of anthelminthic treatment presumptively in the second trimester in addition to iron and folate supplementation (IFAS) where there is high maternal anaemia prevalence [6, 8].

ITN use and IPTp administration during pregnancy are associated with reduced peripheral and placental parasitaemia, maternal anaemia, risk of fetal loss and increased mean birth weight [9–17]. Three or more doses of IPTp improved birthweight and reduced preterm birth risk [18–20]. It is also estimated that if up to 80% of pregnant women reporting for ANC received one dose of IPTp-SP, about 56,000 cases of LBW would have been averted in the WHO African region in 2019 [5]. There also is continued evidence that de-worming during pregnancy reduces the risk of maternal anaemia and improves birth weight and child survival [21, 22].

In Ghana, MiP control interventions have been implemented through the ANC system for over two decades through the collaborative effort of the National Malaria Control Programme (NMCP) and the Reproductive and Child Health department of the Family Health division of the Ghana Health Service (GHS). During routine ANC, interventions such as ITN distribution, IPTp-SP administration under DOT, IFAS, de-worming and giving of health advice including dietary advice for improving anaemia are implemented [23, 24]. Over the years, the uptake of these interventions has seen steady increases. In 2018, 97% of pregnant women made at least one contact with ANC and 85% made four or more visits compared to 90% and 69% respectively in 2003. IPTp-SP coverage of 3 or more doses has more than doubled from 27% in 2008 to 61% in 2019` while ITN use has increased from 3% in 2003 to 49% in 2019 [25–31] (Table 1). These have occurred within the context of decreasing global trends of malaria infection [32] confirmed by a commensurate downward trend of malaria infection in pregnancy prevalence in Ghana [33]. However, not much is known about how these improvements in MiP interventions uptake and reduction in malaria infection prevalence have impacted pregnancy outcomes in the country. To assess the impact of MiP control, the percentage LBW singleton livebirths by parity and percentage of screened women with severe anaemia in 3^rd^ trimester by gravidity are recommended indicators [34].

**Table 1:** Trends in uptake of antenatal care interventions for MiP control in Ghana

	DHS 2003	DHS 2008	MICS 2011	DHS 2014	MIS 2016	MHS 2017	MICS 2018	MIS 2019
ANC from skilled provider	90%	95%		97%		96%	97%	97%
≥ 4 ANC visits	69%	78%		87%		89%	85%	
IPTp 1 + coverage		58%		83%	85%			91%
IPTp 2 + coverage	1.5%	44%	65%	68%	78%			80%
IPTp3 + coverage		28%		39%	60%		52%	61%
ITN use	3%	27%	33%	43%	50%			49%
Iron supplementation (at least 90 days)	40%	42%		59%				

This study therefore aims at describing changes in the prevalence of maternal anaemia and LBW over the last decade by analysing national level data from the District Health Information Management System version II (DHIMS-II) of Ghana. The trends in MiP interventions uptake will also be described and related to these pregnancy outcomes. The findings will throw more light on MiP interventions implementation levels and subsequent pregnancy outcomes in the country, tracking progress towards national goals and inform national policy reviews to further improve MiP pregnancy outcomes.

## Methods

### Study design, area and data source

This was a descriptive study utilizing secondary data from Ghana’s DHIMS II database over a 10-year period from 2012 to 2021. DHIMS II is an integrated internet-based electronic database of aggregated health facility-based data on health services provided nation-wide [35].

Ghana, a sub-Saharan African country located in West Africa, has a total population of 30.8 million; 50.7% females and a total fertility rate of 3.745 births per woman [36]. It is endemic for malaria, being the leading cause of outpatient health facility visits. Malaria accounted for 34% of all cases seen at the Outpatient’s Department (OPD), 19%% of admissions and 2% of total deaths with pregnant women constituting 3.9% of total suspected cases of malaria reported to the OPD in 2017 [37].

The country is divided into three malaria epidemiological zones with varying transmission intensities; the northern Guinea savannah zone covering the northern regions of Ghana with intense and seasonal transmission (and some pockets of perennial transmission in areas of irrigation projects), the transitional forest zone in the middle of the country with perennial and intense transmission and the coastal savannah zone along the coast of the Atlantic Ocean [33, 38]. Malaria infection in pregnancy is highest in the northern Guinea zone, followed by the middle transitional zone and lowest in the coastal savannah zone [33].

There are currently 16 administrative regions, the last 6 being couched from bigger regions in 2018. Each region is divided into metropolitan areas, municipalities or districts depending on population sizes. For health services, metropolitan areas, municipalities and districts are further subdivided into sub-metropolitan areas, sub-municipalities and sub-districts. The regions, metropolis/municipalities/districts and sub-metropolis/sub-municipalities/sub-districts are managed by Regional, Metropolitan/Municipal/District and Sub-metropolitan/Sub-municipal/Sub-district Health Management teams, representatives of the GHS to enhance delivery, supervision and reporting of health services. Health services are delivered through tertiary level (teaching hospitals), secondary level (regional hospitals) and primary level (district/municipal hospitals, health centres and Community-based health planning services (CHPS) compounds) facilities. The health facilities are mostly public but are supported by private, faith-based, traditional and alternative service providers [35]. At all the health facilities (primary to tertiary), primary data of maternal health services is captured manually into paper-based registers, forms and books which are then summarized monthly onto nationally pre-designed forms for further imputing into the DHIMS II data-base either at the district or sub-district level [39]. This enables timely access to health information by health managers and policy makers at the health facility, district, regional and national levels for tracking progress of health service delivery to inform adequate planning, monitoring and evaluation purposes.

### Study variables

Formal permission was sought from the GHS to use the DHIMS II data regarding maternal health services. Based on the aim of this study and availability of data, variables of interest for which data was extracted into an excel spreadsheet included yearly total numbers of:

1. pregnant women visiting the ANC clinic for the first time or at booking (registrants),
2. ANC visits made per woman during pregnancy,
3. pregnant women with maternal anaemia (Hb < 11.0 g/dl) and severe maternal anaemia (Hb < 7 g/dl) at booking and at 36 weeks gestation,
4. LBW (birth weight < 2.5 kg) babies, LBW babies for multiparous and primiparous women,
5. live births,
6. pregnant women receiving 1–5 doses of IPTp, ITN and IFAS for 3 months and 6 months,
7. pregnant women suspected of malaria infection and those testing positive and
8. pregnant women screened and those testing positive for syphilis and HIV.

### Data analysis

The data obtained from the DHIMS II was analysed descriptively and presented as averages and percentages in tables and graphs, first for the whole country and then per zones using Microsoft Excel 365. The regions of Ghana were grouped into three geographical zones to mimic the ecological and malaria epidemiological zones [33, 38] as follows: Northern/savannah zone comprised of the Upper West, Upper East, North East, Northern and Savannah regions; Middle/forest zone comprised of Bono East, Brong Ahafo, Ahafo, Ashanti, Eastern, Western North and Oti regions and the Southern/coastal zone comprised of the Western, Central, Greater Accra and the Volta regions.

Per the definitions in Table 2 below, the various indicators for the years under review were computed. The total number of expected pregnancies was computed as 4% of the estimated yearly population size of Ghana [40]. The country-wide trends over the 10-year period for anaemia and severe anaemia at booking and at 36 weeks gestation, total LBW prevalence and by primiparous and multiparous women; uptake of IPTp-SP and HIV and syphilis infection prevalence were depicted graphically (values in Additional file 1: Table S1) while those for number of ANC visits, IFA supplementation, ITN distribution and malaria test positivity rate were presented in a table. The trends in anaemia, LBW and IPTp-SP uptake were further analysed at geographical zone level, compared with the national averages and depicted graphically or in a table (values in Additional file 1: Table S2).

**Table 2:** Definition of indicators for MiP control and pregnancy outcomes

Indicator	Definition	Numerator	Denominator
ANC Coverage	Proportion of pregnant women receiving antenatal care during pregnancy (at least once)	Total number of antenatal registrants in a specified period	Total number of expected pregnancies of the catchment area within the specified period
ANC 4^+^ Visit	Proportion of pregnant women making at least 4 ANC visits	Number of pregnant women in a specified period making at least 4 ANC visits	Total number of antenatal registrants within the specified period
Average ANC Visit per client	Average number of ANC visits made by all of women delivering in the specified time period	Total number of antenatal attendances (all ANC clients) within the specified period	Total number of antenatal registrants in a specified period
IPTp1-5 uptake	Proportion of pregnant women who received their SP doses 1–5	Number of pregnant women who had their IPT-SP 1–5 at the ANC in a specified period	Total number of antenatal registrants within that specified period
Anaemia (Hb < 11 g/dl) at registration	Proportion of pregnant women anaemic at registration	Number of pregnant women with Hb < 11 g/dl at the time of registration in a specified period	Total number of pregnant women whose Hb were checked at registration within the specified period
Anaemia (Hb < 11 g/dl) at 36 weeks (term)	Proportion of pregnant women anaemic at 36 weeks gestation	Number of pregnant women with Hb < 11 g/dl at 36 weeks in a specified period	Total number of pregnant women whose Hb were checked at 36 weeks within the specified period
Syphilis/HIV infection (among pregnant women)	Proportion of ANC attendees who were screened and tested positive for syphilis/HIV	Number of pregnant women screened for syphilis/HIV in the specified time period who tested positive	Number of pregnant women who were tested for syphilis/HIV in the specified time period
Women receiving IFA for 3 months or 6 months	Proportion of pregnant women receiving IFA for 3 months (90 days) or 6 months (180 days)	Total number of pregnant women receiving IFA for 3 months or 6 months in a given period	Total ANC registrants in the given period
LBW	Proportion LBW	Number of live infants weighing < 2500 g at birth in a specified time period	Total number of live births (with birth weight recorded) in the specified time period
LBW in primips or multips	Proportion of LBW born to primips (women at first delivery) or multips (women at 2^nd^ or more deliveries)	Number of live infants weighing < 2500 g at birth born to primips or multips in a specified time period	Total number of live births (with birth weight recorded) in the specified time period
Malaria test positivity rate	Proportion of pregnant women with suspected malaria who tested positive during ANC	Number of pregnant women suspected of malaria infection who tested positive	Total number of pregnant women with suspected malaria who tested for malaria in a specified time period
ITN distribution	The proportion of ANC registrants given an ITN	Number of ANC registrants given an ITN	Total number of ANC registrants in a specified same period

Source: Ghana Health Service Health Information Management System Standard Operating Procedures, 2020; Maternal and Child Survival Programme Malaria in Pregnancy Monitoring and Evaluation brief, 2020 [88, 89]

## Results

Data extracted covered the period 2012 to 2021. Not all variables of interest had data covering the entire period. Data capture for IPTp 4 and 5, suspected malaria and syphilis infection testing started in 2014 while that for number of doses of IFAS delivered was from 2017.

### Maternal anaemia

**Fig. 1:**
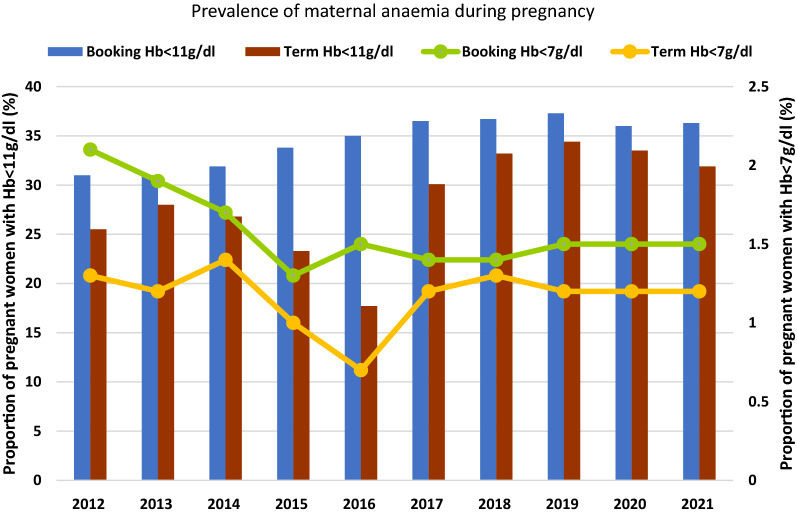
Prevalence of maternal anaemia at booking ANC and at term pregnancy over a ten-year period

The country-wide prevalence of maternal anaemia at booking and at 36 weeks gestation are shown in Fig. 1. There has been a gradual increase in the proportion of women reporting with anaemia over the years under review. Anaemia prevalence at booking increased from 31.0% in 2012 to 36% in 2021 while that at term increased from 25.5% in 2012 to 31.9% in 2021. There was however a noticeable decrease in the prevalence of anaemia at term from 28.0% in 2013 to 17.7% in 2016 but this was not sustained over the following years. The prevalence of anaemia at term was persistently lower than that at booking over the study period. Prevalence of severe anaemia for both multiparous and nulliparous women was just under 2.0% in the last decade, marginally reducing to close to 1.5% in the multiparous women and 1.2% in nulliparous women.

**Fig. 2:**
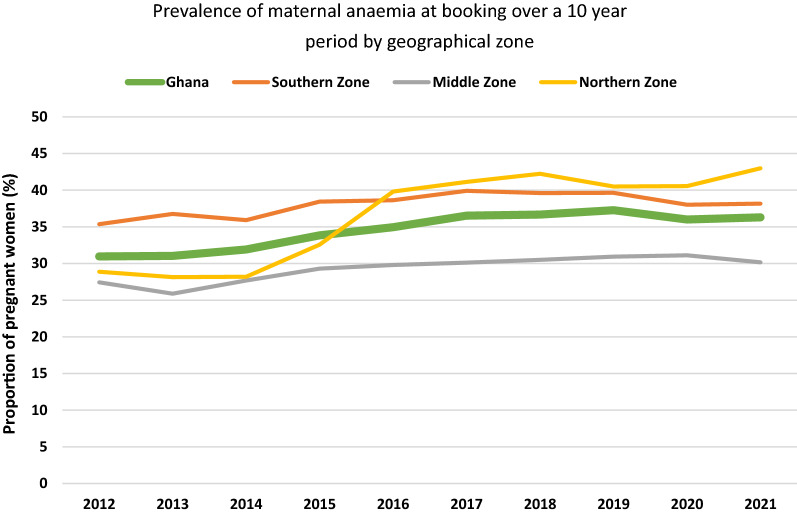
Prevalence of maternal anaemia at booking ANC visit by geographical zone over a ten-year period

**Fig. 3:**
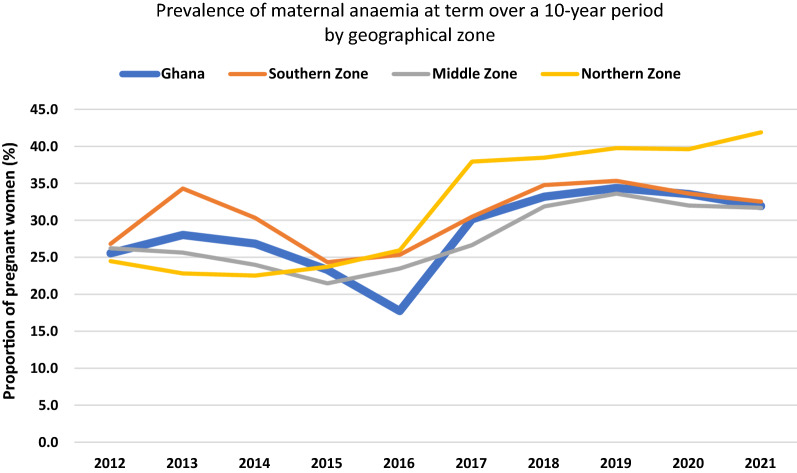
Prevalence of maternal anaemia at term pregnancy by geographical zone over a ten-year period

From Figs. 2 and 3 (and Additional file 1: Fig. S1), the prevalence of anaemia at booking and at term in the Middle zone was the least and largely remained below the national prevalence while that for the Southern zone remained largely above the national prevalence over the study period. Between 2012 and 2015, the prevalence of anaemia (booking and term) in the Northern zone was below the national prevalence but shot up above the national figures by 2016 and remained highest in the country. By 2021, anaemia prevalence at booking ANC visit was 43%, 38.3% and 32.3% in the Northern, Southern and Middle zones respectively while that at term was 41.9%, 32.5% and 31.7% respectively.

### Low birth weight

**Fig. 4:**
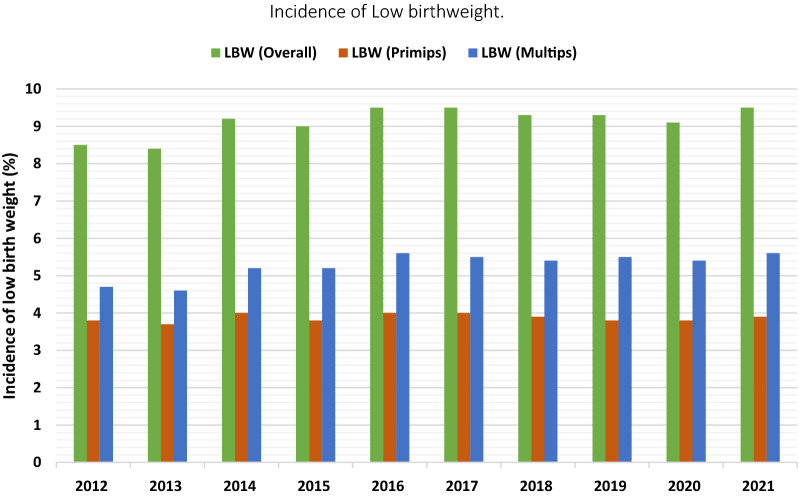
Incidence of low birth weight over a ten-year period

The incidence of LBW gradually increased from 8.5% to 9.5% across the country over the decade. This increase seemed to derive from multiparous women rather than primiparous women. While the proportion of LBW from primiparous women hovered around 4%, that of multiparous women marginally increased from 4.7% to 5.6% as depicted in Fig. 4.

**Fig. 5:**
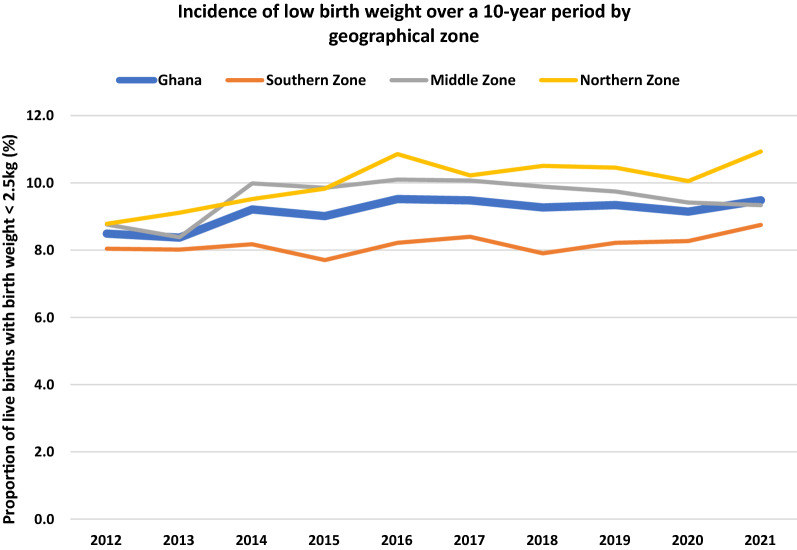
Incidence of LBW by geographical zone over a ten-year period

Comparing LBW incidence in the geographical zones with the national incidence across the study period, LBW incidence in the Northern zone was persistently higher and remained the highest in the country from 2015 while that of the Southern zone remained lower than the national averages and was lowest in the country. LBW incidence in the Middle zone seemed to fluctuate, rising above the national average and the Northern zone incidence by 2014 but declining to remain below that of the Northern zone to just below national average by 2021 (Fig. 5). As at 2021, LBW incidence in the Northern, Middle and Southern zones were 10.9%, 9.3% and 8.7%, respectively.

### Uptake of ANC interventions

#### ANC visits

Table 3 below shows the trend of pregnant women’s ANC visits over the period under review. On average, a pregnant woman made at least 4 ANC visits during pregnancy between 2012 to 2017, rising to at least 5 visits averagely by 2021. Over 90% of the women made at least one contact with ANC from 2012 to 2013 but this figure declined gradually to 78% by the end of 2021. The proportion of women making at least 4 ANC visits however gradually increased from 77% in 2012 to 82% in 2021.

**Table 3:** ANC visits, ITN distributed and IFA doses received by pregnant women from 2012 to 2021

	Year									
ANC indicator	2012	2013	2014	2015	2016	2017	2018	2019	2020	2021
Average number of ANC visits per woman	4.1	4.0	4.2	4.2	4.2	4.4	4.8	4.9	4.9	5.4
ANC coverage	93.5	91.2	88.2	84.8	83.9	80.9	79.0	77.3	79.0	77.7
ANC 4^+^ Visit	77.1	72.7	76.0	75.3	75.9	73.7	74.7	75.3	74.0	82.1
ITN distribution	4.1	11.3	40.0	33.7	72.3	77.3	82.0	83.8	92.1	93.3
Percentage women receiving IFAS for 3 months						43.0	78.8	85.2	87.1	89.7
Percentage women receiving IFAS for 6 months						25.5	55.6	57.0	62.0	61.8

#### IPTp-SP uptake

**Fig. 6:**
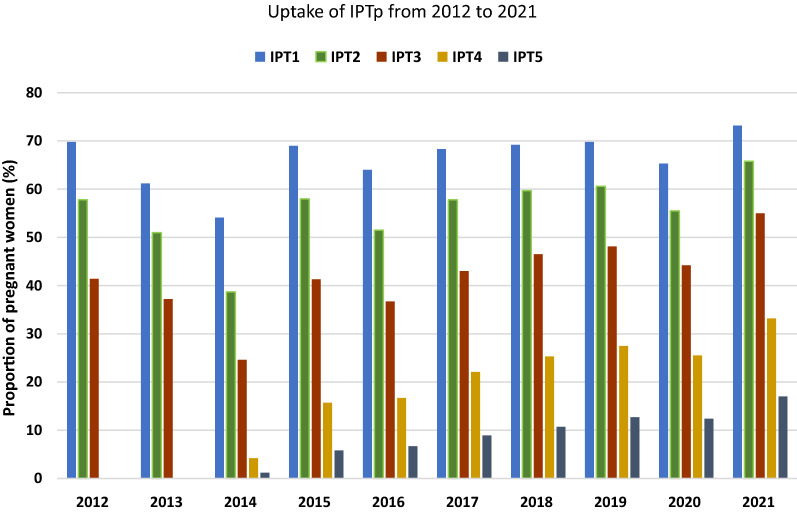
Uptake of IPTp-SP over a ten-year period

Overall, IPTp-SP uptake for all the doses improved over the years (Fig. 6) although there were noticeable dips in 2013, 2014 and 2020. The total number of women receiving at least 3 doses of SP gradually increased. In 2021, 55.0% of women received at least 3 doses of IPTp-SP compared to 41.4% in 2012. IPTp 5 uptake also saw a gradual increase from just 1.2% in 2014 to 17.0% in 2021. The trend of IPTp-SP uptake within the geographical zones for the 1^st^ to 5^th^ doses seemed to follow that of the national prevalence over the study period. However, there seemed to be some fluctuations in uptake in the Northern zone but generally this seemed the lowest compared to the other zones in most of the years under review (Additional file 1: Fig. S2, Table S2).

#### ITN distribution

ITN distribution to the pregnant women saw a dramatic upward trend from a very low 4.1% in 2012 to 93.3% in 2021 (Table 3). Remarkable improvement was seen from 2015 to 2016 where the uptake more than doubled from 33.7% to 72.3% and then continued to rise steadily until 2021.

#### Iron and folate supplementation

The total number of women receiving IFAS has much increased. The available data showed that the proportion of pregnant women receiving IFAS for 3 and 6 months more than doubled from 43.0% to 89.7% and from 25.5% to 61.8%, respectively over a relatively short period from 2017 to 2021 (Table 3).

### Infections in pregnancy

**Fig. 7:**
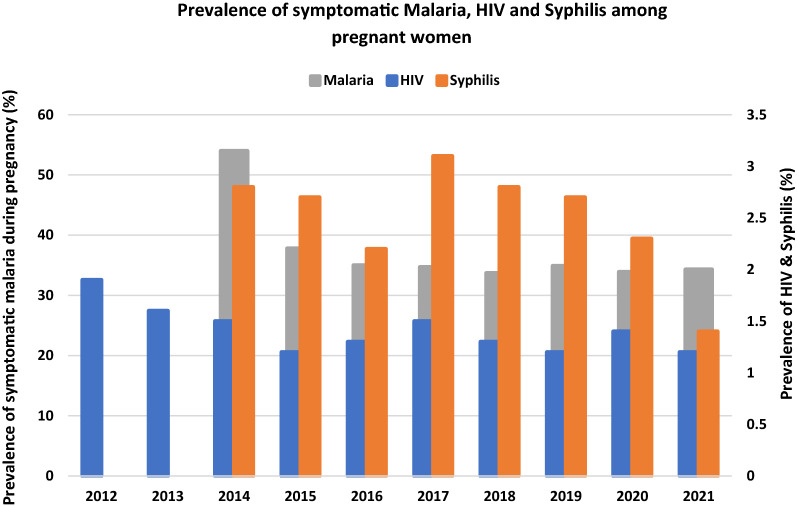
Prevalence of symptomatic malaria, HIV and Syphilis infection over a 10-year period

Malaria test positivity, syphilis and HIV infections prevalence are shown in Fig. 7 below. Malaria test positivity rate declined over the years under study from 54.0% in 2014 to 34.3% in 2021. The prevalence of HIV and syphilis infection in pregnancy decreased slightly over the study period. HIV infection prevalence declined from 1.9% in 2012 to 1.2% in 2021 and syphilis infection from 2.8% in 2014 to 1.4% in 2021.

## Discussion

This study utilized available data from Ghana’s DHIMS II database to describe national trends of maternal anaemia and LBW prevalence, and uptake of MiP control interventions from 2012 to 2021. The prevalence of maternal anaemia at booking increased marginally from 31.0% to 36.3% and that at term also increased from 25.5% to 31.9% over the study period although there was a study decline from 2013 to 2016 showing some possible intervention effect. Similarly, LBW prevalence increased marginally from 8.5% to 9.5%. ANC attendance, IPTp-SP uptake, ITN distribution and IFAS saw appreciable increases while malaria, HIV and syphilis infections among the pregnant women decreased over the study period.

More than 80% of pregnant women made more than 4 ANC visits by 2021, IFAS more than doubled between 2017 and 2021 (Table 3) and IPTp-SP uptake of 3 or more doses also increased by more than 10 percentage points over the study period (Fig. 6). Increasing ANC attendance increases pregnant women’s opportunity to receive more doses of IPTp-SP [41–43] as has been demonstrated by this study. There was a gradual increase in all doses of IPTp received by the pregnant women while the proportion of women making 4 or more ANC visits also gradually increased over the study period (Fig. 6, Table 3).

An upward trend of anaemia prevalence at term pregnancy and LBW was observed despite improving levels of IPTp uptake, IFAS and ANC visits among the women. It was also observed that the percentage decrease in anaemia from booking to term was only marginal (averagely about by 5 percentage points) (Fig. 1). Could it be possible that optimum thresholds for uptake of the interventions have been reached at the current levels of malaria transmission for any more effect on LBW and maternal anaemia prevalence to be seen? In the 1990’s to very early 2000’s when malaria transmission was higher and uptake of interventions minimal, higher prevalence of more than 18% LBW were reported [44–46]. Also, higher maternal anaemia prevalence of more than 60% were recorded across all trimesters of pregnancy between 2004 to 2010 [47, 48]. There is hence little doubt that MiP interventions have been effective in improving birth weight and Hb levels in the past, but this may no longer be applicable under current prevailing malaria transmission and intervention uptake levels.

It could also be argued that much higher intervention uptake levels than what is being reported here are necessary for any further effect to be seen at the current malaria transmission levels. IPTp 1 uptake struggled to reach above 70% during the past decade (Fig. 6), while IPTp 3 uptake was only 55% by 2021. These figures fell short of NMCP’s target of 80% pregnant women receiving 3 doses of IPTp-SP) by 2020 [49]. Missed opportunities to receiving IPTp-SP are still a challenge and have been attributed to several implementation challenges and maternal factors [50–52]. Additional LBW deliveries could be averted in Africa, even in the absence of ITN use, should all pregnant women attending ANC clinics receive IPTp-SP [53]. Confirmed malaria infection during pregnancy in symptomatic women also reduces IPTp-SP uptake as such women should be treated for malaria using other medicines instead of giving IPTp-SP during ANC [24]. There was a decline in malaria test positivity rate from 54.0% in 2012 to 34.3% in 2021 (Fig. 7). Similar decreasing trends of malaria infection have been reported among children under 5 years [27–29, 31] and all-age out-patients in the country [54]. However, the current rate of 1 in every 3 pregnant women suspected of malaria infection testing positive is quite alarming. While these women contribute to lowering IPTp-SP uptake, it presupposes that vector control measures are not being fully adhered to. ITN distribution saw a dramatic upward trend to almost every pregnant woman receiving one at booking ANC visit by 2021 (Table 3) but ownership does not always translate into use. For example, in 2019, 83.3% pregnant women received ITN (Table 3) but only 49% used them [31]. Mathematical modelling has proven that increasing coverage of ITN alone is not enough but increasing the usage of ITN increases their protective efficacy and hence reduces the incidence of uncomplicated malaria cases [55]. Thus, it may be prudent that while the NMCP persists in its efforts aimed at improving malaria control intervention implementation during ANC, there should be intensified social and behaviour change communication to encourage ITN use among pregnant women.

Pregnant women’s adherence to IFAS could also help explain the trends observed. A systematic review of studies conducted in SSA reported adherence to IFAS to be 39.2% [56]. Similarly in Ghana only 40% to 60% of pregnant women adhere to IFAS [57–60]. Hence, increasing supply during ANC does not necessarily mean increasing intake of IFAS among the women. This is also evidenced by the fact that prevalence of iron deficiency (ID) and iron deficiency anaemia (IDA) increase in the third trimester compared to the first trimester values [61, 62] despite iron supplementation. The pregnant women may not be taking the IFAS as expected causing fetal demands to outweigh supply and hence depletion of stores as pregnancy progresses. On the other hand, these studies [61, 62] also reported less than 20% prevalence of ID and less than 10% IDA at booking or in first trimester, a sign that possibly, iron deficiency may not be as prevalent as was reported before. The above prevalence of ID and IDA are mirrored in a recent nation-wide survey among women in reproductive age (WIRA), who eventually transition into pregnancy, where ID and IDA of 13.7% and 8.9% respectively were found [63]. In this same study [63], 4 in 10 anaemic WIRA were iron deficient confirming ID to be an important contributor to anaemia. However, it can also be deduced that almost 6 in 10 WIRA with anaemia is attributable to other causes which need equal attention, especially during pregnancy.

An interesting observation was made comparing the prevalence of anaemia and LBW incidence within the geographical zones over the study period. Anaemia prevalence and LBW incidence were highest in the Northern zone from 2016 to 2021 (Figs. 2, 3 and 5), where the highest malaria infection in pregnancy prevalence in the country has also been reported [33], and where there seemed to be the lowest uptake of IPTp uptake (Additional file 1: Fig. S2) but the same cannot be said for the Middle and Southern zones. Malaria infection in pregnancy prevalence is reported to be the lowest in the Southern zone [33], yet maternal anaemia prevalence at booking and at term seemed higher in this zone compared to the Middle zone (Figs. 2 and 3) where malaria infection in pregnancy is higher but IPTp uptake similar to that of Middle zone (Additional file 1: Fig. S2). LBW incidence however was lower in the Southern zone compared to the Middle zone over the decade. In consequence, the prevalence of maternal anaemia and incidence of LBW across the zones in the country seemed not to correlate with the malaria infection in pregnancy and uptake of IPTp patterns reported. This observation thus seems to buttress the fact that other causes of LBW and anaemia in pregnancy ought to be sought for.

A very complex and multifactorial aetiology of maternal anaemia and LBW has been documented aside malaria and ID. In Ghana, studies have shown that helminths and their co-infection with malaria, schistosomiasis, HIV, chronic hepatitis B infection and syphilis substantially increase the risk of maternal anaemia, preterm and small for gestational age infants [64–69]. This study reported HIV and syphilis infections prevalence to be below 2% by 2021 but their contributions to maternal anaemia and LBW may not be ignored.

Nutritional factors such as maternal undernutrition, being underweight just before pregnancy, inadequate weight gain during pregnancy and practices such as ‘pica’ are associated with increased risk of maternal anaemia and LBW [70–74]. Various maternal demographic and behavioural factors like age, socio-economic status, marital status, educational level, rural residence, ANC seeking-behaviour, late ANC booking, grand multigravidity and multiparity influence maternal anaemia and LBW [74–78] while environmental factors like the use of unsafe water and charcoal for cooking, exposure to heat and the burning of garbage during pregnancy are also identified risk factors [77, 79, 80]. Indeed, genetic factors like being a female neonate and small or short stature of the woman are also associated with LBW [71, 76, 81].

In Ghana, the contribution of haemoglobinopathies and G6PD deficiency to maternal anaemia and LBW may be a concern. Possible haemolysis from oxidative states in sickle cell patients and following IPTp administration in G6PD women may further compound maternal anaemia. Sickle cell anaemia prevalence of under 1%, homozygous or thalassaemia trait of 4.4% in WIRA [63] and G6PD prevalence (partial and full defect) of 0.8% up to 19.3% in pregnant women [10, 82, 83] have been reported in the country. However, G6PD’s effect on maternal anaemia after IPTp intake could be negligible [83]. Other morbidities like hypertensive disorders of pregnancy are also associated with intrauterine growth restriction, preterm and LBW babies [84–86].

With so many identifiable and complex interplay of risk factors and the current observed trends, it is about time a critical look is taken to identify the major contributing factors to maternal anemia and LBW in our setting apart from malaria and ID. Studies that will quantify the contributions of these factors and their level of public health significance are urgently needed. This will enable appropriate and evidence-based strategies to be developed and implemented to achieve effective intervention control.

This study was not without limitations. DHIMS II data may be prone to data quality issues. Nonetheless, data validation teams at facility level supervised by the DHMTs assure high quality DHIMS II data and completeness and accuracy of data on maternal services of above 90% have been documented [39, 87]. Also, DHIMS II data is aggregated thus analysis at the individual women’s level could not be done to contribute to understanding the observed trends. Disaggregation of data including that of anaemia by gravidity would enhance future impact assessments of MiP control interventions. This current study described ecological zone trends in IPTp-SP uptake, maternal anaemia prevalence and LBW incidence but further studies are needed at the district and regional levels for targeted approaches to MiP control and maternal anaemia and LBW prevention. The strength of this study, however, is that it involves a large data set across the whole country and thus gives a national overview of MiP control and its subsequent impact on pregnancy outcomes. This is possibly the first time such an overview study describing MiP control intervention implementation and pregnancy outcomes has been conducted at the national level in Ghana.

## Conclusions

Maternal anaemia and LBW prevalence saw an apparent upward trend over the last decade despite the observed improving implementation of MiP control interventions. There is still room for improvement in the current levels of MiP interventions uptake for which the NMCP should not relent in their efforts at reaching their set targets. However, it is also possible the upper threshold of uptake of MiP interventions has been reached under the prevailing malaria transmission and infection rates and thus no further impact of the interventions was seen. Maternal anaemia prevalence and LBW incidence appeared to be highest in the Northern zone of the country, but the Middle and Southern zones appeared to have mixed patterns in the prevalence of maternal anaemia and incidence of LBW. The complex and multi-factorial aetiologies of anaemia in pregnancy and LBW need thorough investigation and quantification to inform the development of effective prevention programmes for policy and practice.

## Supplementary Information

**Additional file 1: Figure S1.** Prevalence of maternal anaemia at term pregnancy by geographical zone over a ten-year period. **Figure S2.** Trends in IPTp-SP doses 1-5 uptake by ecological zones over a 10-year period. **Table S1.** Trends in IPTp uptake, anaemia, malaria test positivity, LBW, HIV and syphilis prevalence from 2012 to 2021. **Table S2.** Trends in anaemia, LBW and IPTp uptake from 2012 to 2021 by ecological zones.

## Data Availability

The data that support the findings of this study are available from the Ghana Health Service but restrictions apply to the availability of these data, which were used under permission for the current study, and so are not publicly available. Data are however available from the authors upon reasonable request and with permission of the Ghana Health Service.

## Abbreviations

ANC: Antenatal care
DHIMS II: District Health Information Management system version 2
DHMT: District health management team
DHS: Demographic and health survey
DOT: Directly observed treatment
GHS: Ghana health service
Hb: Haemoglobin
HIV: Human immuno-deficiency virus
ID: Iron deficiency
IDA: Iron deficiency anaemia
IFAS: Iron and folate supplementation
IPTp-SP: Intermittent preventive treatment of malaria in pregnancy using sulfadoxine-pyrimethamine
ITN: Insecticide-treated net
LBW: Low birth weight
MHS: Maternal health survey
MICS: Multiple indicator cluster survey
MiP: Malaria in pregnancy
MIS: Malaria indicator survey
NMCP: National Malaria Control Programme
SSA: Sub-Saharan Africa
WHO: World Health Organization
WIRA: Women in reproductive age
