# Supplementary material for: Malaria in pregnancy control and pregnancy outcomes: a decade’s overview using Ghana’s DHIMS II data
Source: Malar J. 2022 Oct 27;21:303. doi: 10.1186/s12936-022-04331-2 (PMC9615308; doi:10.1186/s12936-022-04331-2)
Supplement: Supplementary file 1 — Additional file 1: Figure S1. Prevalence of maternal anaemia at term pregnancy by geographical zone over a ten-year period. Figure S2. Trends in IPTp-SP doses 1-5 uptake by ecological zones over a 10-year period. Table S1. Trends in IPTp uptake, anaemia, malaria test positivity, LBW, HIV and syphilis prevalence from 2012 to 2021. Table S2. Trends in anaemia, LBW and IPTp uptake from 2012 to 2021 by ecological zones. [file 12936_2022_4331_MOESM1_ESM.docx]

Additional file 1: Figure S1: Prevalence of maternal anaemia at term pregnancy by geographical zone over a ten-year period.

*The line for Ghana overall anaemia prevalence for this graph was generated using averages of the values of the three zones for each year.


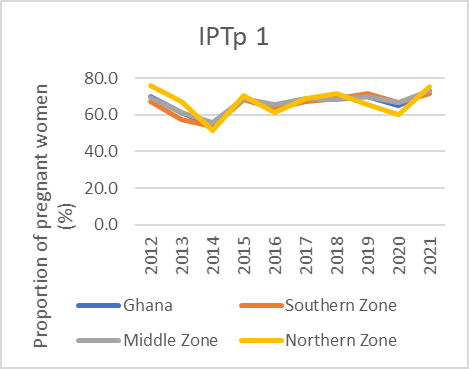

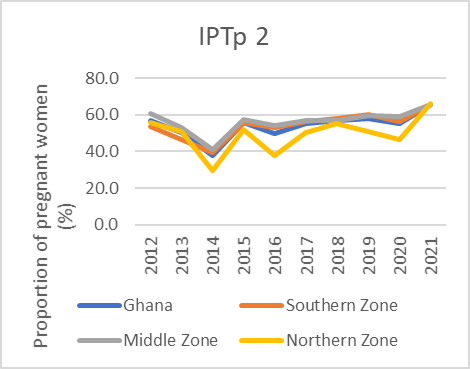

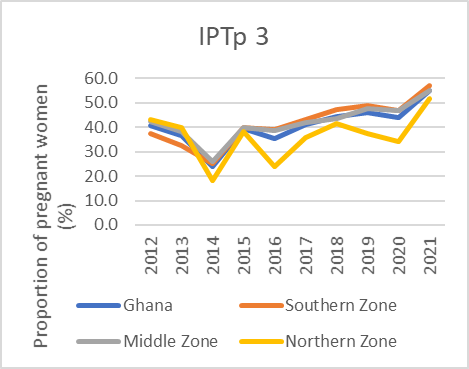

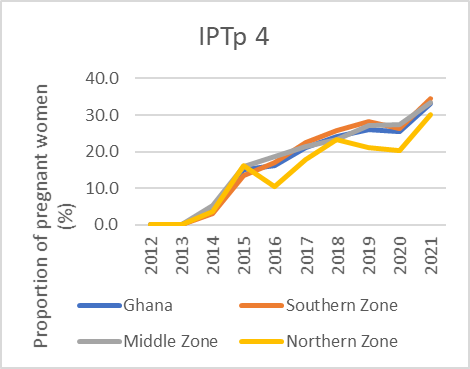

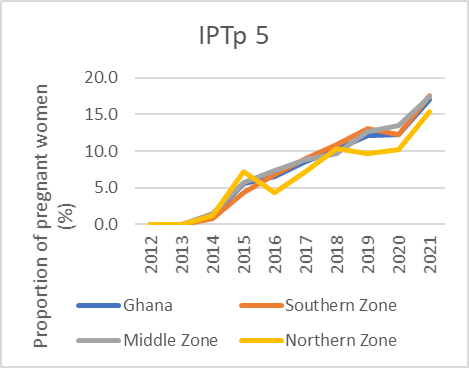


Additional file 1: Figure S2: Trends in IPTp-SP doses 1-5 uptake by ecological zones over a 10-year period

Additional file 1: Table S1: Trends in IPTp uptake, anaemia, malaria test positivity, LBW, HIV and syphilis prevalence from 2012 to 2021

|  | **Year** | | | | | | | | | |
| --- | --- | --- | --- | --- | --- | --- | --- | --- | --- | --- |
| **ANC indicator (%)** | **2012** | **2013** | **2014** | **2015** | **2016** | **2017** | **2018** | **2019** | **2020** | **2021** |
| IPTp1 uptake | 69.8 | 61.2 | 54.1 | 69.0 | 64.0 | 68.3 | 69.2 | 69.8 | 65.3 | 73.2 |
| IPTp 2 uptake | 57.8 | 51.0 | 38.7 | 58.0 | 51.5 | 57.8 | 59.7 | 60.6 | 55.5 | 65.8 |
| IPTp 3 uptake | 41.4 | 37.2 | 24.6 | 41.3 | 36.7 | 43.0 | 46.5 | 48.1 | 44.2 | 55.0 |
| IPTp 4 uptake |  |  | 4.2 | 15.7 | 16.7 | 22.1 | 25.3 | 27.5 | 25.5 | 33.2 |
| IPTp 5 uptake |  |  | 1.2 | 5.8 | 6.7 | 8.9 | 10.7 | 12.7 | 12.4 | 17.0 |
| Anaemia (Hb < 11g/dl) at registration | 31.0 | 31.0 | 31.9 | 33.8 | 35.0 | 36.5 | 36.7 | 37.3 | 36.0 | 36.3 |
| Anaemia (Hb < 11g/dl) at 36 weeks (term) | 25.5 | 28.0 | 26.8 | 23.3 | 17.7 | 30.1 | 33.2 | 34.4 | 33.5 | 31.9 |
| Severe anaemia (Hb < 7g/dl) at registration | 2.1 | 1.9 | 1.7 | 1.3 | 1.5 | 1.4 | 1.4 | 1.5 | 1.5 | 1.5 |
| Severe anaemia (Hb < 7g/dl) at 36 weeks (term) | 1.3 | 1.2 | 1.4 | 1.0 | 0.7 | 1.2 | 1.3 | 1.2 | 1.2 | 1.2 |
| LBW (total) | 8.5 | 8.4 | 9.2 | 9.0 | 9.5 | 9.5 | 9.3 | 9.3 | 9.1 | 9.5 |
| LBW in primips | 3.8 | 3.7 | 4.0 | 3.8 | 4.0 | 4.0 | 3.9 | 3.8 | 3.8 | 3.9 |
| LBW in multips | 4.7 | 4.6 | 5.2 | 5.2 | 5.6 | 5.5 | 5.4 | 5.5 | 5.4 | 5.6 |
| HIV infection | 1.9 | 1.6 | 1.5 | 1.2 | 1.3 | 1.5 | 1.3 | 1.2 | 1.4 | 1.2 |
| Syphilis infection |  |  | 2.8 | 2.7 | 2.2 | 3.1 | 2.8 | 2.7 | 2.3 | 1.4 |
| Malaria test positivity rate |  |  | 54.0 | 37.8 | 35.0 | 34.7 | 33.7 | 34.9 | 33.9 | 34.3 |

Additional file 1: Table S2: Trends in anaemia, LBW and IPTp uptake from 2012 to 2021 by ecological zones

|  |  |  | **Year** | |  |  |  |  |  |  |  |
| --- | --- | --- | --- | --- | --- | --- | --- | --- | --- | --- | --- |
| **ANC indicator** | **Zone** | **2012** | **2013** | **2014** | **2015** | **2016** | **2017** | **2018** | **2019** | **2020** | **2021** |
| Anaemia (Hb < 11gm/dl) at registration | Ghana | 31.0 | 31.0 | 31.9 | 33.8 | 35.0 | 36.5 | 36.7 | 37.3 | 36.0 | 36.3 |
|  | Southern Zone | 33.8 | 36.6 | 36.6 | 38.3 | 37.7 | 39.2 | 39.4 | 39.6 | 38.4 | 38.3 |
|  | Middle Zone | 30.6 | 29.1 | 29.7 | 32.0 | 32.9 | 33.3 | 33.2 | 33.4 | 32.9 | 32.3 |
|  | Northern Zone | 28.9 | 28.1 | 28.2 | 32.6 | 39.8 | 41.1 | 42.2 | 40.5 | 40.6 | 43.0 |
| Anaemia (Hb < 11gm/dl) at 36 weeks (term) | Ghana | 25.5 | 28.0 | 26.8 | 23.3 | 17.7 | 30.1 | 33.2 | 34.4 | 33.5 | 31.9 |
|  | Southern Zone | 26.8 | 34.3 | 30.3 | 24.3 | 25.3 | 30.5 | 34.8 | 35.3 | 33.6 | 32.5 |
|  | Middle Zone | 26.2 | 25.6 | 24.0 | 21.5 | 23.5 | 26.6 | 31.9 | 33.6 | 32.0 | 31.7 |
|  | Northern Zone | 24.5 | 22.8 | 22.5 | 23.7 | 25.9 | 37.9 | 38.5 | 39.8 | 39.6 | 41.9 |
| LBW | Ghana | 8.5 | 8.4 | 9.2 | 9.0 | 9.5 | 9.5 | 9.3 | 9.3 | 9.1 | 9.5 |
|  | Southern Zone | 8.0 | 8.0 | 8.2 | 7.7 | 8.2 | 8.4 | 7.9 | 8.2 | 8.3 | 8.7 |
|  | Middle Zone | 8.8 | 8.4 | 10.0 | 9.9 | 10.1 | 10.1 | 9.9 | 9.7 | 9.4 | 9.3 |
|  | Northern Zone | 8.8 | 9.1 | 9.5 | 9.8 | 10.9 | 10.2 | 10.5 | 10.5 | 10.1 | 10.9 |
| IPT 1 | Ghana | 69.8 | 61.2 | 54.1 | 69.0 | 64.0 | 68.3 | 69.2 | 69.8 | 65.3 | 73.2 |
|  | Southern Zone | 67.1 | 57.6 | 53.7 | 68.6 | 63.4 | 67.4 | 69.2 | 71.9 | 66.5 | 71.8 |
|  | Middle Zone | 69.4 | 61.5 | 55.8 | 68.7 | 65.9 | 68.8 | 68.2 | 70.0 | 66.9 | 73.2 |
|  | Northern Zone | 76.1 | 67.1 | 51.3 | 70.4 | 61.5 | 69.0 | 71.5 | 65.9 | 60.4 | 75.8 |
| IPT 2 | Ghana | 57.0 | 50.2 | 37.8 | 55.6 | 49.9 | 55.3 | 57.0 | 57.8 | 55.3 | 65.8 |
|  | Southern Zone | 53.6 | 46.5 | 39.3 | 55.9 | 53.0 | 56.5 | 58.2 | 60.1 | 56.4 | 65.6 |
|  | Middle Zone | 60.6 | 53.2 | 40.9 | 57.4 | 54.0 | 56.9 | 57.0 | 59.6 | 59.1 | 65.7 |
|  | Northern Zone | 56.1 | 50.9 | 29.6 | 51.9 | 37.6 | 50.5 | 55.0 | 50.7 | 46.6 | 66.3 |
| IPT 3 | Ghana | 40.8 | 36.6 | 24.0 | 39.6 | 35.5 | 41.1 | 44.4 | 45.9 | 44.1 | 55.0 |
|  | Southern Zone | 37.6 | 32.7 | 25.3 | 39.8 | 39.0 | 43.2 | 47.2 | 49.1 | 46.9 | 57.0 |
|  | Middle Zone | 42.4 | 38.3 | 26.0 | 40.0 | 38.9 | 42.2 | 43.4 | 47.7 | 46.9 | 55.0 |
|  | Northern Zone | 43.3 | 39.8 | 18.2 | 38.5 | 24.0 | 35.8 | 41.7 | 37.6 | 34.4 | 51.8 |
| IPT4 | Ghana | 0.0 | 0.0 | 4.0 | 15.1 | 16.2 | 21.1 | 24.2 | 26.2 | 25.4 | 33.2 |
|  | Southern Zone | 0.0 | 0.0 | 3.1 | 13.4 | 17.1 | 22.5 | 25.7 | 28.2 | 26.4 | 34.6 |
|  | Middle Zone | 0.0 | 0.0 | 5.2 | 15.9 | 18.6 | 21.6 | 23.3 | 27.2 | 27.4 | 33.5 |
|  | Northern Zone | 0.0 | 0.0 | 3.6 | 16.3 | 10.5 | 18.0 | 23.3 | 21.1 | 20.4 | 30.2 |
| IPT 5 | Ghana | 0.0 | 0.0 | 1.1 | 5.5 | 6.5 | 8.5 | 10.2 | 12.1 | 12.3 | 17.0 |
|  | Southern Zone | 0.0 | 0.0 | 0.8 | 4.3 | 6.8 | 8.9 | 10.8 | 13.1 | 12.3 | 17.6 |
|  | Middle Zone | 0.0 | 0.0 | 1.5 | 5.7 | 7.4 | 8.8 | 9.7 | 12.6 | 13.5 | 17.4 |
|  | Northern Zone | 0.0 | 0.0 | 1.1 | 7.2 | 4.4 | 7.3 | 10.3 | 9.6 | 10.2 | 15.4 |
